# Prevalence and risk factors of falls among community-dwelling older people: results from three consecutive waves of the national health interview survey in Taiwan

**DOI:** 10.1186/s12877-020-01922-z

**Published:** 2020-12-09

**Authors:** Yih-Jian Tsai, Ping-Yen Yang, Yi-Ching Yang, Mau-Roung Lin, Ying-Wei Wang

**Affiliations:** 1 Health Promotion Administration, Ministry of Health and Welfare, Taipei, Taiwan; 2 Department of Geriatrics and Gerontology, College of Medicine, National Cheng-Kung University and Hospital, Tainan, Taiwan; 3 Graduate Institute of Injury Prevention and Control, College of Public Health, Taipei Medical University, Taipei, Taiwan; 4 Hualien Tzu Chi Hospital, Tzu Chi University and Hospital, Hualien, Taiwan

**Keywords:** Prevalence, Risk factors, Fall prevention, Elderly

## Abstract

**Background:**

An aging society incurs great losses due to fall-related injuries and mortalities. The foreseeable increased burden of fall-related injury among older people requires a regular nationwide study on the fall epidemic and prevention strategies.

**Methods:**

The fall epidemic was examined using data from three consecutive waves of the National Health Interview Survey (2005, 2009, and 2013). Common explanatory variables across these surveys included sociodemographic factors (age, sex, and difficulty in performing activities of daily living (ADL) or instrumental ADL), biological factors (vision, comorbidities, urinary incontinence, and depressive symptoms), and behavioral risk factors (sleeping pill use, and frequency of exercise). After the univariate and bivariate analyses, the prevalence of falls was investigated using multiple linear regression models adjusted for age group, sex, and year of survey. A multivariate logistic regression model for falls with adjustments for these common explanatory variables was established across three waves of surveys. The effect of fall prevention programs was examined with the effect size in terms of age-specific and sex-specific prevalence of falls and fall-related hospitalization rates during 2005 and 2009.

**Results:**

For each survey, there were consecutively 2722; 2900; and 3200 respondents with a mean age of 75.1, 75.6, and 76.4 years, respectively. The multiple linear regression model yielded a negative association between the prevalence of falls and year of survey. Several sociodemographic and biological factors, including female sex, difficulty in performing one basic ADL, difficulty in performing two or more instrumental ADLs, unclear vision, comorbidities, urinary incontinence, and depressive symptoms, were significantly associated with falls. In contrast to the universal positive effect on the prevalence of falls among older adults, the effect size of fall-related hospitalization rates revealed a 2% relative risk reduction only for those aged 65–74 years, but deteriorated for those aged 75–84 (− 10.9%).

**Conclusion:**

Although the decline in fall prevalence over time supports existing fall intervention strategies in Taiwan, the differential prevention effect and identification of risk factors in older people suggest the necessity of adjusting fall prevention programs.

**Supplementary Information:**

The online version contains supplementary material available at 10.1186/s12877-020-01922-z.

## Background

The annual prevalence of falls has increased by age, from 28 to 35% for people aged ≥65 years to 32 to 42% for those aged > 70 years [1]. As the burden of fall-related injury increased annually by 21.1% between 1990 and 2013, falls among older people have become a global health concern. Furthermore, the burden of fall-related injuries reached a new peak of 27.5 million disability-adjusted life years in 2013 [2]. Falls have not only created tremendous costs in high-income countries [3–6], but the increased rate of fall-related injuries has gradually affected the health and ability of older individuals in low- and middle-income countries to perform daily tasks. Main risk factors for falls are categorized into four dimensions: biological, behavioral, environmental, and socioeconomic factors [1]. Given the multifactorial etiology of falls [7], the prevalence and risk factors of falls or fall-related injuries vary by age of the target population, country, outcomes, and covariates measured, etc. For the need of setting up a sound evidence base of fall prevention policy, increasing number of countries include fall-related issues in national surveys.

Analysis of the 1997–2010 National Health Interview Survey (NHIS) in the United States revealed that 61.9% of all fall-related injuries among older women occurred indoors while occurred 32.8% outdoors. Sedentary older individuals usually experience fall-related injuries indoors, while older people with a high level of physical activity experience falls outdoors [8]. While the multivariable logistic regression (MLR) models showed that both sexes shared some risk factors for falls, such as chronic health conditions and severe pain, sex-specific risk factors were also identified. These risk factors include incontinence and frailty for women, and depressive symptoms, advanced age, and inability to maintain full-tandem stance for men [9].

In low- and middle-income countries, the pooled prevalence of past-year fall-related injuries was 4% across six countries (China, Ghana, India, Mexico, Russian Federation, and South Africa) [10]. However, among these countries and Indonesia, the most common risk factors of fall-related injuries in older adults is having two or more comorbidities, and the less common risk factors include depression, sleeping problems, and poor cognitive function [10, 11].

In southern Taiwan, falls accounted for 60% of trauma admissions of older patients in 2009 and 2013 [12]. Analysis of data from the 1996 and 1999 Taiwan Longitudinal Study on Aging demonstrated that the overall prevalence of falls was 19.5%. Furthermore, the risk of falling was higher among individuals with the following characteristics: female sex, having a disability, reduced activities of daily living (ADL) function, depressive symptoms, using a cane or a walker but still walking well, and not wearing glasses but not seeing clearly [13]. In response to concerns about the growing health care burden from fall injury in companion with an ageing population, a multifactorial fall risk awareness program was launched in 2004 and later integrated into Health Promotion Programs for the Elderly in 2009 [14]. In this study, we attempt to investigate the prevalence and risk factors for falls among community-dwelling older people in 2005, 2009, and 2013, and the effect of fall prevention programs on selected fall-related outcomes, to better document fall prevention policies.

## Methods

### Study subjects and data collection

Data of persons aged ≥65 years were collected from three consecutive waves of the NHIS (2005, 2009, and 2013) in Taiwan. The design and sampling strategies used for the 2005 NHIS were described in a previous report [15]. Those who could not tolerate an interview because of self-perceived physical weakness, difficult hearing, deafness, dumbness, or mental problems were replaced by proxy as stipulated, rather than being excluded from the study. Participants were drawn at a probability proportional to the size of the older population using a multiple-stage, stratified, systematic sampling design. In summary, we drew consecutively 187, 164, and 168 of the 358 townships or districts nationwide for each wave of surveys. These townships or districts drawn were further divided into 53 strata according to their geographic location, population distribution, and preceding interview experience. Within each stratum selected, sampling stages varied by the degree of urbanization. Two-stage sampling was conducted for those within high urbanization strata, first with neighborhood unit and then persons. Three-stage sampling was conducted for those within the moderate urbanization strata, first with villages, followed by neighborhood unit and persons, and for those within rural or remote strata, first with townships/districts, followed by neighborhood unit and persons. Between April 2005 and August 2005, data were collected from 2727 persons (85.5%) among 3188 eligible subjects aged ≥65 years using face-to-face questionnaire interviews for the NHIS. The 2009 and 2013 NHIS surveys were conducted in a similar manner to the 2005 NHIS survey using a Computer Assisted Personal Interview. These surveys had response rates of 88.2% (2904/3294) and 82.8% (3204/3868), respectively. After four or five respondents who did not specify their fall experience in each consecutive wave of survey in 2005, 2009, and 2013 were excluded, 2722; 2900; and 3200 respondents were included in the analysis. Data quality was assured through standardization of the questionnaire administration process and auditing.

### Outcomes and explanatory variables

A fall was defined as “an event of falling down that occurs while one stands up, sits down, gets into bed, or walks, etc., regardless of its underlying causes or resting on a same or lower level.” Each participant that experienced a fall was required to answer (Yes/No) if he/she had experienced a fall, regardless of frequency, in the past year.

Common explanatory variables that were chosen for the correlation analyses with fall data included sociodemographic factors (age, sex, and difficulty in performing activities of daily living (ADL) or instrumental ADL), biological factors (vision, comorbidities, urinary incontinence, and depressive symptoms [16]), and behavioral risk factors (sleeping pill use, and frequency of exercise) (Additional file 1: Table S1).

### Statistical analyses

The statistical software package used to conduct analyses was SAS version 9.3. For each wave of survey results, data were weighted to correct for the probability of multistage sampling. Univariate analyses were used to examine the frequency distribution of each explanatory variable. The chi-square test was used to compare the risk of falling across each explanatory variable. The prevalence of falls was defined according to the proportion of participants who experienced at least a fall in the population at risk in the past year and was further stratified by age group and sex to obtain stable estimates of age- and sex-specific prevalence. A multiple linear regression model that was adjusted for age group, sex, and year of the survey was used to examine the time-dependent changes in the age- and sex-specific prevalence of falls. A MLR model was established and adjusted for all the aforementioned common explanatory variables to investigate the independent association between each explanatory variable and the odds of having experienced a fall in the past year. Statistical significance was set at α = 0.05. The time-dependent trends of age- and sex-specific prevalence of falls were then compared with those of the overall, sex-specific, and age-specific fall-related hospitalization rates between 2003 and 2009 [17]. The effect of fall prevention programs during 2005 and 2009 was examined with the effect size of relative risk reduction [18] in terms of age-specific and sex-specific prevalence of falls and fall-related hospitalization rates derived from a previous report [17]. For each specific rate, the effect size was defined as the rate difference during 2005 and 2009, divided by the observed rate in 2005, as calculated in the following.

$$ \mathrm{Es}=100\ast \left({\mathrm{r}}_0-{\mathrm{r}}_1\right)/{\mathrm{r}}_0 $$

Where, Es stands for the effect size (%). Es > 0 means a positive effect, while Es < 0 means a negative effect.

r_0_ was the observed rate in 2005, when the baseline measurement was taken. It was plausible because the multifactorial fall risk awareness program was just a pilot study in 2004 for a later distribution.

r_1_ was the observed rate in 2009, which was regarded as the effect measurement for comparison based on data availability.

## Results

The characteristics of the participants varied across the three consecutive waves of national surveys in 2005 and 2013 (Table 1). Characteristics that increased by survey included the proportions for those aged 80–84 years, aged ≥85 years, and using sleeping pills (from 10.9, 12.6, to 14.9%). Accordingly, the mean age (±SD) of respondents increased from 75.1 ± 6.0, 75.6 ± 6.3, to 76.4 ± 6.5 years, respectively. The proportion of two or more comorbidities also escalated from 31.2, 32.5, to 39.1%. On the contrary, those characteristics that decreased by survey included the proportions for unclear vision (from 25.6, 24.9, to 20.3%), irregular exercise (from 6.1, 5.0, to 3.5%), and depressive symptoms (from 28.6, 23.6, to 20.4%).

**Table 1:** Characteristics of study subjects by number of participants and prevalence of falls during survey years

Characteristics	2005				2009				2013			
	No. of participants		Prevalence of falls and *p*-value		No. of participants		Prevalence of falls and *p*-value		No. of participants		Prevalence of falls and *p*-value	
	*N* = 2722	%	n	%	*N* = 2900	%	n	%	*N* = 3200	%	n	%
Total	2722	100.0	579	21.3	2900	100.0	565	17.5	3200	100.0	528	16.5
Mean age (±SD, year)	75.1	±6.0			75.6	±6.3			76.4	±6.5		
Age				0.002				0.017				0.004
65–69	868	31.9	147	16.9	877	30.2	131	14.0	852	26.6	110	12.1
70–74	743	27.3	161	20.9	726	25.0	141	18.5	866	27.1	135	16.2
75–79	619	22.7	141	23.2	653	22.5	131	16.4	654	20.4	115	17.9
80–84	329	12.1	88	28.1	388	13.4	96	23.0	503	15.7	99	18.7
85+	163	6.0	42	26.1	256	8.8	66	21.3	325	10.2	69	24.3
Sex				< 0.001				< 0.001				0.224
Male	1346	49.4	221	16.5	1252	43.2	198	14.1	1523	47.6	227	15.3
Female	1376	50.6	358	26.2	1648	56.8	367	20.6	1677	52.4	301	17.5
ADL difficulty				< 0.001				< 0.001				< 0.001
None	2324	85.4	419	18.1	2382	82.2	399	15.2	2658	83.1	379	13.8
1 task	74	2.7	33	45.9	109	3.8	33	29.0	112	3.5	38	44.5
≥ 2 tasks	322	11.8	127	39.4	406	14.0	133	28.4	427	13.4	111	27.2
IADL difficulty				< 0.001				< 0.001				< 0.001
None	1574	58.1	232	15.0	2275	79.4	371	14.8	1943	61.5	244	11.9
1 task	361	13.3	67	19.0	105	3.7	28	25.1	349	11.0	57	16.0
≥ 2 tasks	775	28.6	278	35.7	486	17.0	161	29.1	868	27.5	216	27.5
Use of sleeping pills use				0.005				0.022				0.009
No	2422	89.1	496	20.4	2521	87.4	468	16.7	2715	85.1	422	15.4
Yes	297	10.9	83	28.6	363	12.6	94	22.9	477	14.9	106	22.3
Vision				< 0.001				0.002				< 0.001
Clear	772	32.1	129	17.4	815	32.0	109	12.8	974	34.2	109	11.1
Average	1020	42.4	184	17.7	1097	43.1	206	17.0	1296	45.5	189	14.3
Unclear	615	25.6	166	28.1	635	24.9	159	22.2	579	20.3	146	27.7
Frequency of exercise				0.018				0.001				< 0.001
None	1246	45.8	298	24.2	1370	50.0	311	21.2	1524	50.4	301	20.3
Irregular	167	6.1	34	19.3	138	5.0	17	13.0	107	3.5	20	13.4
Regular	1306	48.0	247	19.1	1233	45.0	196	13.8	1394	46.1	170	12.6
Comorbidities				< 0.001				0.051				0.022
0	879	38.5	116	12.2	942	33.5	153	14.2	884	28.6	104	12.4
1	690	30.2	131	19.2	957	34.0	185	17.6	995	32.2	175	16.4
≥ 2	712	31.2	187	28.5	916	32.5	202	19.6	1207	39.1	218	18.5
Urinary incontinence				< 0.001				0.002				< 0.001
No	2066	76.1	384	18.8	2603	90.2	485	16.5	2544	79.9	367	14.0
Yes	649	23.9	194	29.6	284	9.8	77	26.0	640	20.1	156	25.5
Depressive symptoms				< 0.001				< 0.001				< 0.001
No	1715	71.4	282	16.3	1999	76.4	297	13.6	2229	79.6	282	12.5
Yes	686	28.6	198	30.0	617	23.6	193	27.7	570	20.4	139	24.9

Note: *p*-value < 0.05 using the chi-square test indicates a statistically significant fall risk across each explanatory variable. The prevalence of falls was estimated by weighing according to sampling probability proportional to the population size. *SD* standard deviation

Table 2 shows that the proportions of some morbidities increased in 2005 and 2013 for hypertension (from 43.1, 49.1, to 53.3%), diabetes (from 17.8, 19.3, to 22.5%), hyperlipidemia (from 23.3, 24.0, to 26.8%), and asthma (from 5.2, 5.3, to 6.3%), while the proportions of other morbidities fluctuated. With regard to the prevalence of falls, those older adults who had any of the selected chronic conditions tended to have a higher risk of falls than those who had none.

**Table 2:** Distribution of falls across selected chronic conditions during the year of survey

Characteristics	2005				2009				2013			
	No. of participants		Prevalence of falls and *p*-value		No. of participants		Prevalence of falls and *p*-value		No. of participants		Prevalence of falls and *p*-value	
	*N* = 2722	%	n	%	*N* = 2900	%	n	%	*N* = 3200	%	n	%
Hypertension				< 0.001				0.002				0.078
No	1514	56.9	277	17.4	1475	50.9	247	14.7	1482	46.5	221	14.7
Yes	1145	43.1	279	25.9	1421	49.1	317	20.3	1708	53.5	304	17.9
Diabetes				< 0.001				0.032				0.131
No	2181	82.2	424	19.7	2337	80.7	431	16.5	2471	77.5	386	15.7
Yes	473	17.8	134	28.4	560	19.3	134	21.5	717	22.5	137	18.9
Hyperlipidemia				< 0.001				0.979				0.014
No	1847	76.7	327	17.9	2159	76.0	421	17.2	2290	73.2	354	14.8
Yes	562	23.3	149	26.6	682	24.0	127	17.3	839	26.8	155	19.9
Stroke				< 0.001				0.921				< 0.001
No	2493	92.4	504	20.1	2687	92.8	518	17.4	2918	91.3	448	15.0
Yes	206	7.6	67	35.5	210	7.2	45	17.7	277	8.7	79	32.4
Transient ischemic attack				< 0.001				0.037				0.008
No	2447	90.3	488	20.2	2702	93.7	514	16.9	2838	89.6	444	15.4
Yes	263	9.7	89	32.5	182	6.3	48	24.6	329	10.4	74	24.1
Asthma				0.050				0.001				0.648
No	2561	94.9	533	20.9	2744	94.7	519	16.9	2993	93.7	496	16.6
Yes	140	5.2	38	29.1	155	5.3	46	29.6	201	6.3	30	15.0
Kidney disease				< 0.001				0.464				0.006
No	2375	91.1	467	19.5	2666	92.3	501	17.2	2884	90.4	456	15.5
Yes	232	8.9	72	33.9	223	7.7	60	19.7	308	9.6	70	24.3

Note: *p* < 0.05 using the chi-square test indicates a statistically significant fall risk across each explanatory variable. The prevalence of falls was estimated by weighing according to sampling probability proportional to the population size

Table 3 reveals the risk of falls varied by each variable and survey. Risk of falls was higher in women and those with advanced age, with the exception of women in 2013 and those aged ≥85 years between 2005 and 2009. Older people who had urinary incontinence and depressive symptoms and used sleeping pills tended to have a higher risk of falls. Moreover, older adults were more likely to have a fall with a higher number of comorbidities and of IADL difficulty, but not with that of ADL difficulty. Notably, there was a gradient of protective effect from risk of falls by vision quality, with a moderate risk for average vision and a higher risk for unclear vision. Older people who took regular exercise had their fall risk reduced by 26 to 43% in 2005 and 2013.

**Table 3:** Univariate logistic regression analyses for falls by the year of survey

Covariate (reference)	2005	2009	2013
	OR 95% CI	OR 95% CI	OR 95% CI
Age (65–69)			
70–74	1.30 (0.99–1.71)	1.40 (1.06–1.84)	1.40 (1.04–1.89)
75–79	1.48 (1.12–1.96)	1.20 (0.90–1.62)	1.58 (1.15–2.17)
80–84	1.92 (1.39–2.65)	1.83 (1.34–2.51)	1.67 (1.20–2.34)
85+	1.74 (1.14–2.65)	1.66 (1.13–2.42)	2.33 (1.62–3.35)
Sex (male)			
Female	1.80 (1.47–2.21)	1.57 (1.28–1.93)	1.18 (0.95–1.45)
ADL difficulty (none)			
1 task	3.84 (2.34–6.30)	2.29 (1.46–3.60)	5.00 (3.22–7.75)
≧2 tasks	2.93 (2.23–3.85)	2.22 (1.71–2.87)	2.33 (1.77–3.07)
IADL difficulty (none)			
1 task	1.32 (0.96–1.82)	1.92 (1.18–3.11)	1.42 (1.00–2.01)
≧2 tasks	3.14 (2.52–3.90)	2.36 (1.85–3.00)	2.82 (2.24–3.54)
Use of sleeping pills (no)			
Yes	1.56 (1.17–2.08)	1.48 (1.11–1.96)	1.58 (1.22–2.04)
Vision (clear)			
Average	1.02 (0.78–1.33)	1.39 (1.07–1.82)	1.33 (1.01–1.75)
Unclear	1.86 (1.41–2.45)	1.94 (1.44–2.60)	3.07 (2.28–4.13)
Comorbidities (0)			
1	1.71 (1.27–2.30)	1.30 (1.00–1.68)	1.39 (1.03–1.86)
≧2	2.86 (2.15–3.79)	1.47 (1.14–1.91)	1.60 (1.21–2.11)
Urinary incontinence (no)			
Yes	1.82 (1.46–2.26)	1.78 (1.33–2.38)	2.10 (1.67–2.65)
Depressive symptoms (no)			
Yes	2.20 (1.76–2.75)	2.44 (1.94–3.07)	2.32 (1.80–3.00)
Frequency of exercise (none)			
Irregular	0.75 (0.48–1.17)	0.56 (0.34–0.91)	0.60 (0.33–1.10)
Regular	0.74 (0.60–0.91)	0.60 (0.48–0.74)	0.57 (0.45–0.71)

**Fig. 1:**
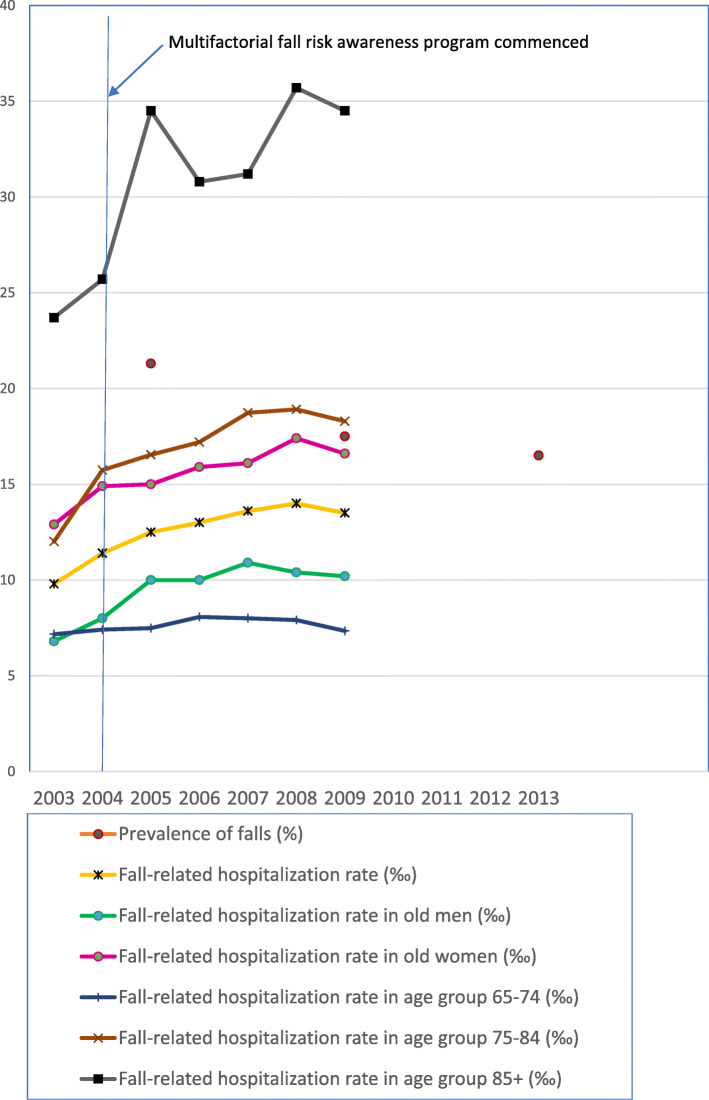
Time trends in the prevalence of falls and fall-related hospitalization rates

As shown in Table 1, the weighted prevalence of falls (and 95% confidence interval) over the previous year gradually dropped from 21.3% (95% CI 19.6–23.1%), 17.5% (95% CI 15.7–19.2%), to 16.5% (95% CI 14.8–18.3%) across three waves of survey. With further adjustment for age, sex, and year of survey accounting for 76% of the total variation in the multiple linear regression model (R^2^ = 0.76 in Additional file 2: Table S2), the age- and sex-specific prevalence of falls presented a decrease rate of 2.61% per year during the period from 2005 to 2013. In contrast to the declining trend of the prevalence rates for falls during the period from 2005 to 2013, an increasing trend was observed for the overall, sex-specific, and age-specific fall-related hospitalization rates between 2003 and 2009 [17]. This trend was especially apparent among older women and individuals aged 75–84 years (Fig. 1).

The overall and sex-specific and age-specific fall-related hospitalization rates from 2003 to 2009 were adopted from Bai [17]. Bai’s fall-related data were retrieved from the inpatient expenditures by admissions (DD) of the longitudinal national health insurance research database (LHID) 2005. This database contained information on patients aged ≥65 years and hospitalized due to fall injuries with diagnostic code E880-E888 of the International Classification of Disease-Clinical Modification (ninth revision), either for external cause codes or for major diagnosis and secondary diagnosis.

Compared with the univariate logistic regression results (Table 3), Table 4 reveals that independent risk factors of falls identified in the MLR models varied with attenuation of adjusted odds ratios (OR) across these surveys. Risk of falls increased 1.4–1.6 times in women (OR 1.64, 95% CI 1.26–2.15 in 2005; OR 1.38, 95% CI 1.09–1.76 in 2009), but not with age. Those risk factors that were independently associated with a higher risk of falls included urinary incontinence (OR 1.42, 95% CI 1.04–1.94 in 2013), depressive symptoms (OR 1.51, 95% CI 1.12–2.03 in 2005; OR 1.77, 95% CI 1.35–2.31 in 2009; OR 1.45, 95% CI 1.06–1.98 in 2013), and having difficulty in performing one ADL (OR 2.39, 95% CI 1.25–4.58 in 2005; OR 2.74, 95% CI 1.55–4.86 in 2013), having difficulty in performing two or more IADLs (OR 1.45, 95% CI 1.00–2.11 in 2005), and unclear vision (OR 1.92, 95% CI 1.36–2.72 in 2013), instead of using sleeping pills. Moreover, a fall-risk gradient was found between having one (OR 1.61, 95% CI 1.16–2.24) and two or more comorbidities (OR 2.41, 95% CI 1.74–3.35) in 2005. Notably, both regular and irregular exercises were not associated with a protective effect from falls.

**Table 4:** Multivariate logistic regression analyses for falls by the year of survey

Covariate (reference)	2005		2009		2013	
Age (65–69)						
70–74	1.16	(0.83–1.62)	1.26	(0.93–1.69)	1.13	(0.81–1.58)
75–79	1.24	(0.87–1.76)	0.87	(0.62–1.22)	1.17	(0.81–1.70)
80+	1.34	(0.88–2.03)	1.35	(0.97–1.89)	1.19	(0.82–1.73)
Sex (male)						
Female	1.64	(1.26–2.15)	1.38	(1.09–1.76)	0.93	(0.72–1.20)
ADL difficulty (none)						
1 task	2.39	(1.25–4.58)	0.44	(0.10–1.87)	2.74	(1.55–4.86)
≥ 2 tasks	1.41	(0.80–2.47)	0.42	(0.09–2.04)	1.14	(0.66–1.95)
IADL difficulty (none)						
1 task	0.76	(0.51–1.14)	1.41	(0.74–2.72)	1.13	(0.77–1.66)
≥ 2 tasks	1.45	(1.00–2.11)	4.56	(0.98–21.23)	1.27	(0.85–1.90)
Use of sleeping pills (no)						
Yes	1.09	(0.75–1.60)	1.06	(0.75–1.49)	1.21	(0.88–1.67)
Vision (clear)						
Average	0.93	(0.69–1.27)	1.25	(0.94–1.65)	1.10	(0.82–1.48)
Unclear	1.09	(0.77–1.55)	1.23	(0.88–1.71)	1.92	(1.36–2.72)
Comorbidities (0)						
1	1.61	(1.16–2.24)	1.16	(0.87–1.54)	1.18	(0.85–1.64)
≥ 2	2.41	(1.74–3.35)	1.19	(0.89–1.61)	1.12	(0.81–1.54)
Urinary incontinence (no)						
Yes	1.09	(0.80–1.49)	1.29	(0.90–1.84)	1.42	(1.04–1.94)
Depressive symptoms (no)						
Yes	1.51	(1.12–2.03)	1.77	(1.35–2.31)	1.45	(1.06–1.98)
Frequency of exercise (none)						
Irregular	1.20	(0.70–2.04)	0.77	(0.46–1.29)	0.87	(0.45–1.69)
Regular	1.12	(0.85–1.49)	0.81	(0.63–1.04)	0.80	(0.62–1.03)

Note: Adjusted odds ratios and 95% confidence interval (OR and 95%CI) are presented for each dummy variable. Variables controlled across three waves of survey in the MLR model included age, sex, developing difficulty in performing ADLs or IADLs, use of sleeping pills, vision, comorbidities, urinary incontinence, depressive symptoms, and frequency of exercise

**Fig. 2:**
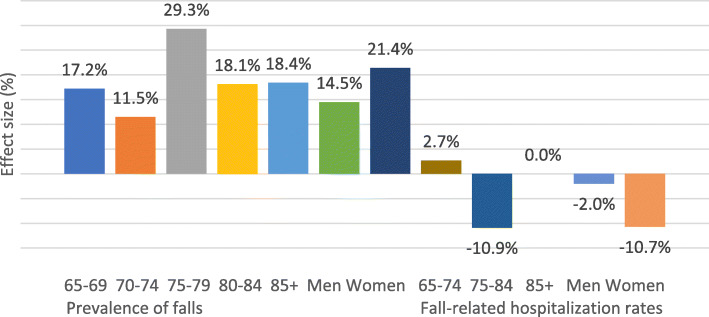
Effect sizes of prevalence of falls and fall-related hospitalization rates during 2005 and 2009

Fig. 2 demonstrates that the effect size varied by age and sex of study population and outcome indicators selected. During 2005 and 2009, the effect size of prevalence of falls presented as 14.5% for old men and 21.4% for old women, and 17.2, 11.5, 29.3, 18.1, and 18.4% respectively for those aged 65–69, 70–74, 75–79, 80–84, and ≥ 85 years. In contrast to the universal positive effect on the prevalence of falls among older adults, the effect size of fall-related hospitalization rates revealed a 2% relative risk reduction only for those aged 65–74 years, but deteriorated for those aged 75–84 (−10.9%), for old men (−2.0%) and old women (−10.7%).

## Discussion

Our study is the first to present the differential effect of fall prevention programs on falls and fall-related hospitalizations. Several significant independent risk factors for falls identified included the following: female sex, difficulty in performing one basic ADL, difficulty in performing two or more instrumental ADLs, unclear vision, comorbidities, urinary incontinence, and depressive symptoms. However, no significant risk of falling was associated with advanced age, use of sleeping pills, and performing regular or irregular exercise. Furthermore, these risk factors and differential prevention effect may help dictate the future direction of fall prevention policies.

Variation of the time-dependent characteristics of the participants by survey reflected more the influence of demographic, epidemiological, and health transitions [19], rather than cross-survey comparability. The increased prevalence of multiple comorbidities may result from a rapidly aging population, earlier detection, and better treatment of disease [20]. Our analyses on the effect sizes indicate that the effect of fall prevention programs varied by age and sex of study population and outcome indicators selected. Successful fall prevention strategies are supposed to encompass the full array of contributing variables or causes over a broad target audience with user-designed strategies [21] and to accomplish a significant risk reduction in falls and fall-related hospitalizations. On one hand, the declining prevalence of falls may reflect the combined effect of these community-based multifactorial fall risk awareness programs mentioned above. On the other hand, despite appearing to serve the wider community-dwelling older people, these programs may benefit the young–old (65 years old) over the old–old (≥75 years) population for prevention from fall-related hospitalizations. The differential effect of these programs can be attributed to the differences in study population and factors influencing implementation. Compared with the community-based prevalence of falls, the fall-related hospitalization rates were estimated on a broader population base including those older people living in long-term institutionalized care. In spite of a small share of the national elderly, these institutionalized older people, who have twice the incidence rate of major injuries of their home-dwelling counterparts [22], were not exposed to these programs. More importantly, four factors which influence implementation of fall prevention programs can be noted, including restricted access [23], prevalence of disability [24], adapting for community, and transforming identities [25]. First, the old–old adults may be more likely to have restricted access to preventive services due to social determinants of health inequality [23], such as financial or geographic barriers and digital divide. Second, as the population is rapidly aging, the functional disability status among older Taiwanese accelerates over time, especially among women and the old–old population. Older women suffer from disproportionately greater levels of disability [24] and are more susceptible to falls and fall-related injuries than their male counterparts (Fig. 1). Third, social and cultural influences are also crucial for individual choice about participation in fall prevention programs [25], such as learning use of assistive devices and uptake of exercise interventions. Fourth, older adults may override risk-averse advice due to their pride and fear of loss of identity of independence and self-esteem [25].

As regards the sociodemographic risk factors for falls that were identified using the MLR models, women had a higher risk of falls than men. Two possible explanations may be applied: (1) they are more liable to osteoporosis and reduced knee muscle strength [26] and (2) they are more susceptible to an indoor fall [27]. The association between difficulties in performing two or more IADLs and an increased risk of falls is compatible with the findings of previous reports [28]. However, there was no corresponding finding among those older adults with difficulty in performing ADLs. A possible explanation is that they were subjected to selective survival [29] and the sample number became too small to obtain a stable OR across the three waves of survey. Another explanation is that those older adults who had difficulty in performing ADLs might be restricted from activity at risk of falls because of existing multiple health problems [30].

Considering biological factors, our finding that older adults with unclear vision had an OR that was twice as high as older adults with clear vision aligns with that in a previous report by Lord [31]. Our finding that having any of the selected chronic conditions was associated with a higher risk of falls is compatible with previous falls and multi-morbidity studies in terms of asthma [32], hypertension [33], diabetes [34], stroke or transient ischemic attack [35, 36], and chronic kidney disease [37, 38]. Moreover, our finding in the fall-risk gradient among older people having one or two or more comorbidities is not only consistent with those of past research [39, 40], but also strengthens the assertion of the additive effect of chronic disease on fall risk [33].

Besides, the 40% higher risk of falls among respondents with urinary incontinence in the 2013 survey was compatible with the conclusions drawn from a previous systematic review [41]. The fact that depressive symptoms were proven to be a significant risk factor of falls might be explained by an intricate bidirectional and self-perpetuating interaction between depression and falls [42].

The main strength of our study is that it has a comparable fall-related questionnaire administered to a large sample size of older adults on a national scale. These factors make it possible to identify the prevalence and risk factors of falls across the three waves of surveys. The national survey data and data of fall-related hospitalizations [17] complemented each other to open up a window of opportunity for us to examine the differential effect of fall prevention programs. However, several limitations of this study are worthy of mention. First, a cross-sectional survey cannot infer a causal relationship between the outcome and explanatory variables. Second, data collection through questionnaire interview might be subject to recall bias, and result in underreporting of falls and non-identification of multiple falls. Additionally, the fall risk among those aged 80 and over, which has shown an increasing time trend in China [39], might be biased with a limited sample size in the survey data. Besides, some older adults were reluctant to verbalize their incontinence because of embarrassment or because of its interference with sexual function. Third, the effect sizes were estimated during too short an observation period, from 2005 to 2009, to indicate long-term variation of fall-related hospitalization rates. Fourth, the association between fall prevention programs and prevention effect could be confounded by other overlapping health promotion programs, such as Community Health Building and Safe Communities [14]. Accordingly, further assessment of risk factor studies on falls have to be conducted with either fall diaries or weekly or monthly follow-up to identify multiple or recurrent falls and minimize reliance on recall of fall events [18, 43]. It is also worthy to conduct further studies to evaluate fall prevention programs in terms of efficiency, effectiveness, and economy [44].

Our study findings imply that a combination of low-risk and high-risk strategies [45] should be adopted to tailor fall prevention programs to people with several different risk factors for falls. Current fall prevention guidelines [46] do not address the potential risk of falls derived from multi-morbidity [32, 33]. Thus, an approach that accounts for adults who have multimorbidity and are prescribed multiple medicines is recommended because such population has a higher risk for adverse events and drug interactions [47]. Considering the projected 2.7-fold growth in the number of hip fractures between 2010 and 2035 [48], the fall epidemic must be surveyed regularly, as the identification of risk factors and differential prevention effect in older people may help with developing individualized fall risk assessments [43] among high-risk seniors.

## Conclusions

This study gives an illustration of using national survey data in conjunction with data of fall-related hospitalizations to investigate the effect of fall prevention programs, in addition to examining the prevalence and risk factors of falls among community-dwelling Taiwanese older people. Although the decline in fall prevalence over time supports existing fall intervention strategies in Taiwan, the differential prevention effect and identification of risk factors in older people suggest the necessity of adjusting fall prevention programs.

## Supplementary Information

**Additional file 1: Table S1.** Definitions of explanatory variables.

**Additional file 2: Table S2.** Multiple linear regression model for the age- and sex-specific prevalence of falls.

## Data Availability

The NHIS datasets used and/or analyzed during the current study were available from the Health and Welfare Data Science Center, Ministry of Health and Welfare, Taiwan, upon regular application.

## Abbreviations

ADL: Activities of daily living
IADL: Instrumental activities of daily living
MLR: Multivariable logistic regression
NHIS: National Health Interview Survey
Es: Effect size of relative risk reduction
HPA: Health Promotion Administration
