# Supplementary material for: Prevalence and risk factors of falls among community-dwelling older people: results from three consecutive waves of the national health interview survey in Taiwan
Source: BMC Geriatr. 2020 Dec 9;20:529. doi: 10.1186/s12877-020-01922-z (PMC7724833; doi:10.1186/s12877-020-01922-z)
Supplement: Supplementary file 1 — Additional file 1: Table S1. Definitions of explanatory variables. [file 12877_2020_1922_MOESM1_ESM.docx]

Table S1 Definitions of explanatory variables

| Variables | Definitions |
| --- | --- |
| Age group | 65-69, 70-74, 75-79, 80-84, and ≥85 years |
| Sex | male, female |
| Difficulty in performing activities of daily living (ADL difficulty) | ADL difficulty was measured in performing six selected ADLs (including bathing, getting dressed, eating, getting in/out of bed (or sitting down/getting up from a chair), walking around the house, and using the toilet). The spectrum of difficulty in realization of each ADL task shifted from “no difficulty,” “somehow difficult,” “very difficult,” to “cannot do it at all.” The sum of difficulties performing ADLs was computed and categorized as none, one, or two or more. |
| Difficulty in performing instrumental activities of daily living (IADL difficulty) | IADL difficulty was measured in performing five selected instrumental ADLs (IADLs; including buying daily needs, managing finances (e.g., performing calculations and paying bills), doing heavy housework, doing light housework (e.g., arranging the living room and washing dishes), and making telephone calls). The spectrum of difficulty in realization of each IADL task shifted from “no difficulty,” “somehow difficult,” “very difficult,” to “cannot do it at all.” The sum of difficulties in performing IADLs was computed and categorized as none, one, or two or more. |
| Vision | Visual ability was classified using the following categories: very clear, clear, average, unclear, very unclear, and blindness, with the additional possibility of wearing glasses. These measurements were then regrouped into three levels of vision quality: clear (including being very clear/clear), average, and unclear (including being unclear/very unclear/blindness). |
| Comorbidity score | Respondents were required to answer (Yes/No/Unknown) whether they experienced any of the following seven chronic conditions: hypertension, diabetes, hyperlipidemia, stroke, transient ischemic attack, asthma, and kidney disease. The comorbidity score was computed using the sum of these chronic diseases, categorized as none, one, or two or more. |
| Urinary incontinence | Urinary incontinence was determined based on a (Yes/No) response to the following question: “During the previous year did you have such an experience of being unable to control urination?” |
| Depressive symptoms | Depressive symptoms were measured with a cutoff point of 8 for the sum score of 10 questions adopted from the original Centers for Epidemiologic Studies Depression Scale [16]). |
| Sleeping pill use | The use of sleeping pills was defined for those respondents who admitted taking sleeping pills usually or regularly in 2005 or at least twice monthly in 2009 or 2013. |
| Frequency of exercise | In 2005 and 2009, data regarding exercise level were collected based on a (Yes/No) response to the question, “During the previous 2 weeks did you participate in any exercise, such as jogging, chuang, or dancing?” This exercise excluded requirement of physical labor in the workplace and/or home. Furthermore, only exercise performed for more than 10 min at a time were counted in these surveys. “Chuang” includes numerous traditional Chinese martial arts such as Tai-Chi and similar activities. The self-reported frequency of exercise was further divided by two to obtain the weekly frequency, equivalent to that recorded in 2013. Respondents were regrouped into the following categories based on their exercise level: none (no exercise), exercising irregularly (twice or less per week), exercising regularly (three or more times per week). |
