# Supplementary material for: Prevalence and risk factors of falls among community-dwelling older people: results from three consecutive waves of the national health interview survey in Taiwan
Source: BMC Geriatr. 2020 Dec 9;20:529. doi: 10.1186/s12877-020-01922-z (PMC7724833; doi:10.1186/s12877-020-01922-z)
Supplement: Supplementary file 2 — Additional file 2: Table S2. Multiple linear regression model for the age- and sex-specific prevalence of falls. [file 12877_2020_1922_MOESM2_ESM.docx]

**Table S2** Multiple linear regression model for age- and sex-specific prevalence of falls

| Variable | Coefficient | 95% CI | p-value |
| --- | --- | --- | --- |
| Age group | 2.33 | (1.48–3.17) | <0.001 |
| Sex | 6.99 | (4.60–9.39) | <0.001 |
| Year of survey | −2.61 | (−4.07 to −1.14) | 0.001 |
| Intercept | 7.48 | (2.06–12.91) | 0.009 |
| R^2^ = 0.76  Adjusted R^2^ = 0.73 | | | |

n = 30 point estimates of age- and sex-specific prevalence of falls. CI = confidence interval.
